# Responsibility and Authority

**Published:** 1908-07-18

**Authors:** 


### Resident Medical Officers' Department.

### RESPONSIBILITY AND AUTHORITY.

The early weeks of his first hospital appointment are full of difficulties and anxieties to the newly-qualified house officer. A new aspect of the medical life, only vaguely realised whilst he was a student, is suddenly opened out to his view, and the process of adjustment, though salutary, is seldom pleasant. Responsibility is now fully tasted for the first time, and the words, diagnosis, treatment, and prognosis suddenly assume wider meanings than they had in the examination hall or in the sheltered atmosphere of student appointments.

The discipline of the first days of medical practice differs widely in hospital and in private. Putting it very broadly, in general practice the novice runs the gauntlet of his patients and their friends, while in hospital he runs the gauntlet of his colleagues. Each is an excellent discipline, and every man should experience both as soon as possible. The general opinion seems to be that at the very beginning of his professional career the medical man gains more from a house appointment than from plunging at once into private practice. There is, of course, no question that every man should obtain hospital experience if he can possibly do so before settling down. Nothing short of urgent private reasons can excuse him from taking at least one house appointment. And it is probably better for him to begin this work immediately after qualifying, and to readjust his methods and ideas to the requirements of general practice at a later date.

Since responsibility is at one and the same time the most trying and the most valuable experience which a hospital appointment affords, it is well to consider it a little more fully. In private practice responsibility is very heavy, and the practitioner is forced to bear it for the most part alone, except when he calls in a colleague for a second opinion. At the same time his authority is great, and he has, or should have, the entire confidence of his patients and their friends. During the greater part of his work there is no one to interfere or to challenge the conduct of his cases. Thus while his responsibility is heavy, it is undivided, and so long as his patients trust him the doctor's authority is supreme.

In hospital, on the other hand, responsibility, although scarcely less heavy, is on a different footing. The house officer certainly has individual responsibilities of various kinds, many of which he has to bear alone. But his responsibility towards his patients is shared by others. In the wards, for instance, it is shared by his superior officer. And the house surgeon himself is responsible to many more people than to his patients alone; to the hospital itself, to his visiting surgeon, to the students under him (if he is at a teaching hospital) and perhaps to some intermediate functionaries as well. His authority again is limited, and often ill-defined. Thus in hospital responsibility and authority are less complete than in general practice, and are far more complicated and nebulous. All this is practically inevitable. Hospitals are corporate institutions, and labour and responsibility and all else must be divided to some extent.

But the practice of different hospitals varies very greatly in this matter. Setting aside the obvious fact that each governing body holds its own views on the proper position and relation to the hospital of the resident medical officers, we may classify the larger hospitals into two broad groups—those hospitals which, in addition to house physicians and house surgeons and resident anæsthetists, possess a senior resident medical officer, or resident assistant physician or surgeon; and those which do not. We have no intention of discussing here the relative advantages and disadvantages to a hospital of having a senior medical man in residence. The question as a whole is a large one, and involves the consideration of many principles. But it is possible to narrow down the matter to the point of view of the house officers themselves.

The house surgeons and house physicians in a large hospital have their greatest responsibility and authority at night time. It is then that emergencies most commonly occur, and then that the visiting staff as a whole is comparatively inaccessible. Circumstances constantly arise which need prompt decision and prompt action. Now, is it or is it not an advantage to the house officers to feel that there is a superior officer always at hand?

What are the points in favour of this practice? In any sudden, grave, or obscure crisis the house surgeon knows that he has reliable help at hand—he may ask for it officially or unofficially, but there it is. And from the constant proximity of the senior R.M.O. he is likely to gain much practical information otherwise not available. There are other advantages, but these will do.

On the other side of the house surgeon's ledger we find considerations of this sort:—Those men who are competent, reliable, cool in emergency, and eager to think and act for themselves, are in theory at least handicapped by the knowledge that on big occasions there is someone in residence with them to share their responsibility and authority. There is always, of course, the visiting physician or surgeon at the other end of the telephone; but that is a very different thing. Again, those house officers who by habit or in the process of mental development are inclined to seek help in difficult positions, and divide the responsibility for action or inaction, are likely to avail themselves too often of the presence of a senior R.M.O. to the detriment of their own character and professional prospects. Hospital appointments are, indeed, schools of character, and self-reliance and decision rather than dexterity and knowledge, are the most valuable lessons that are taught there. Therefore the presence of a senior R.M.O. or resident assistant physician or surgeon, even if his authority is seldom exercised, and his services are seldom requisitioned, is by no means an unmixed blessing to the junior medical staff. Beyond that it is not necessary or desirable for us to intrude.

